# Binary RuO_2_–CuO Electrodes Outperform RuO_2_ Electrodes in Measuring the pH in Food Samples

**DOI:** 10.1021/acsomega.3c00538

**Published:** 2023-03-30

**Authors:** Maryna Lazouskaya, Iuliia Vetik, Martti Tamm, Kiranmai Uppuluri, Ott Scheler

**Affiliations:** † School of Science, Department of Chemistry and Biotechnology, Tallinn University of Technology, Ehitajate tee 5, 19086 Tallinn, Estonia; ‡ Center of Food and Fermentation Technologies (TFTAK), Mäealuse 2/4, 12618 Tallinn, Estonia; § Łukasiewicz Research Network—Institute of Microelectronics and Photonics (Łukasiewicz—IMiF), Kraków Division, ul. Zabłocie 39, 30-701 Kraków, Poland

## Abstract

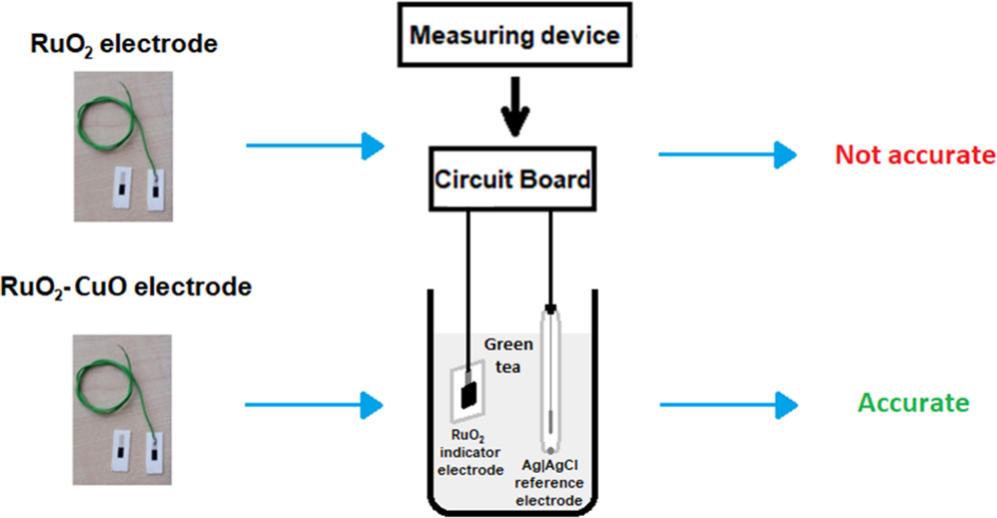

Glass electrodes are the only type of pH-sensitive electrodes currently used in the food industry. While widely used, they have several disadvantages, especially in the areas of brittleness and price. Ruthenium(IV) oxide (RuO_2_) pH electrodes are a well-known alternative to conventional glass electrodes, providing improved durability and lower price. Nevertheless, partial substitution of RuO_2_ with cupric oxide (CuO) would further lower the price and reduce the toxicity of the electrode. In this paper, we present the applicability of RuO_2_–CuO electrodes for pH measurement in food samples. The electrodes were fabricated by screen printing and covered with a protective Nafion membrane. In the experiments with food samples, the RuO_2_–CuO electrodes outperformed RuO_2_ electrodes in measuring the pH with an almost twofold higher rate of accurate measurements. The utilization of CuO for the fabrication of pH electrodes allowed the accurate measurement of pH in a larger variety of food samples without compromising the response time.

## Introduction

Screen-printed electrodes are a widely investigated alternative to bulky and fragile glass electrodes. Screen-printed electrodes are known to be cheap and sensitive and are capable of replacing conventional glass electrodes in pH measurements. Among the different screen-printed electrodes studied for pH measuring applications, ruthenium(IV) oxide (RuO_2_)-based electrodes have shown the best characteristics.^1,2^ RuO_2_-based electrodes are highly sensitive to pH changes over a broad range and are chemically and thermally stable and biocompatible.^1^

Screen-printed electrodes based on RuO_2_ have previously shown excellent pH sensitivity when used in water samples.^3,4^ Screen printing is one of the simplest and cheapest methods for the fabrication of pH-sensitive solid-state electrodes.^1^ It allows the deposition of layers with a thickness on the micrometer scale with excellent mechanical stability and good adherence to various substrates.^3^ Screen-printed RuO_2_ electrodes have close to Nernstian sensitivity and low hysteresis and drift rate in a wide pH range.^1,3^

The sensitivity of RuO_2_ electrodes to the pH of the solution is based on the following reaction (simplified)^2^
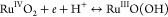
1

The Nernst equation for the reaction 1 allows for a quantitative description
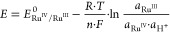
2where *E*^0^ is the standard potential (individual potential of a reversible electrode (in equilibrium) in the standard state), V; *R* is the universal gas constant, 8.314 J/K·mol; *T* is the temperature, K; *n* is the number of electrons participating in the redox reaction; *F* is the Faraday constant, 96485 C/mol; *a*_Ru^IV^_ and *a*_Ru^III^_ are the activities of Ru^IV^O_2_ and Ru^III^O(OH), respectively, mol/L; and *a*_H^+^_ is the activity of H^+^ ions, mol/L.

Considering that the value of the activities of metals is approximately 1 in the solid state, substituting the constants at room temperature (*T* = 22 °C) and replacing −lg *a*_H^*+*^_ with pH, eq 2 takes the following form
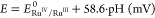
3eq 3 allows the determination of the pH of the solution by measuring the electrochemical potential of the RuO_2_ electrode. Furthermore, in practice, the theoretical (Nernstian) sensitivity value of 58.6 mV/pH might not be observed.^3^ The sensitivity is determined separately for each electrode by determining the electrode potential in several buffer solutions and calibrating the electrode against buffers with known pH values.

**Figure 1:**
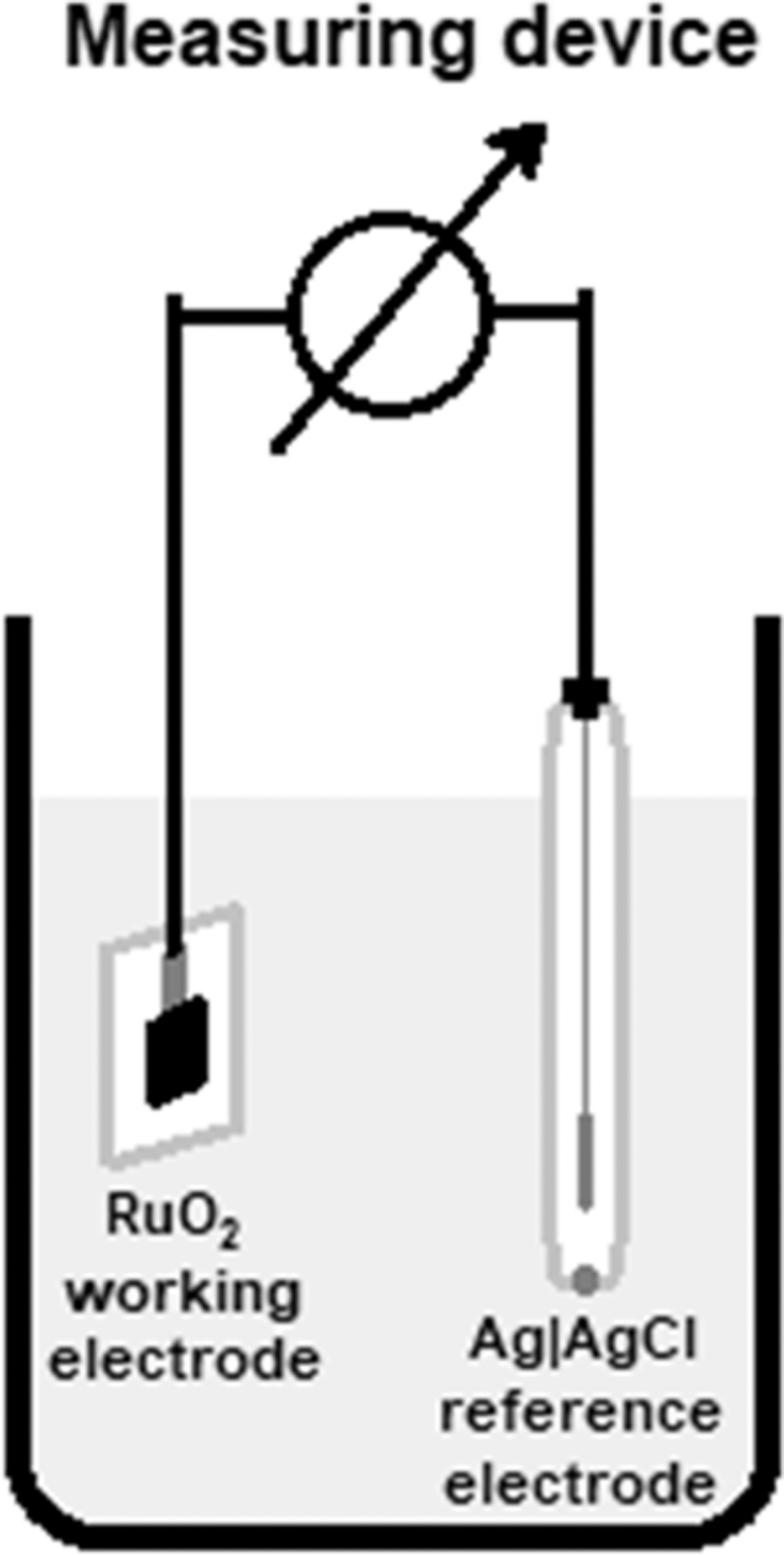
Schematic representation of an electrochemical cell. Primarily, an electrochemical cell for pH measurement consists of (i) a working electrode where the reaction involving H^+^ ions takes place, (ii) a reference electrode that provides a stable and well-known potential, and (iii) a measuring device.

The electrochemical potential of an electrode can be determined by connecting it to an electrochemical cell for potentiometric measurement. Such an electrochemical cell consists of (i) a measuring device—usually a galvanometer or voltmeter, (ii) an electrode of interest, which is called an indicator or working electrode (WE) (in our case—RuO_2_ electrode), and (iii) a reference electrode (RE) with a stable potential that allows determining the potential of the indicator electrode (usually Ag|AgCl electrode) (Figure 1). The measuring device detects the difference in the electrochemical potentials between the indicator and the reference electrode.

At present, the application of screen-printed electrodes for pH measurement in food samples is limited due to the inability of electrodes to perform in complex media (e.g., containing fats or proteins).^5^ Modification of screen-printed electrodes with a Nafion protective membrane was previously demonstrated to improve the performance of RuO_2_ electrodes in milk.^5^ Furthermore, the introduction of the Nafion membrane does not significantly alter the most important electrochemical characteristics of the pH-sensitive RuO_2_ electrode.^5^

However, since ruthenium is a rare element,^6^ it is not the best material to be used for the mass production of pH electrodes. Therefore, to improve the economic and ecological aspects of RuO_2_ electrodes, it is possible to use binary oxides, where a part of RuO_2_ is substituted with another oxide.^4,7−10^ One of the oxides that can be used for this purpose is copper(II) oxide (CuO). Copper(II) oxide is easy to synthesize in various shapes (nanoribbons,^11^ -flowers,^11−13^ -wires,^13^ -rods,^14^ etc.) and sizes;^12−14^ therefore, allowing the surface area to be improved. Due to its high specific surface area, chemical stability, electrocatalytic activity, and low price copper(II) oxide is widely used as an electrocatalyst,^15^ supercapacitor,^16^ photodetector,^16^ and photocatalyst,^16^ sensor for glucose,^13,16^ humidity, and various gases,^16^ such as ethanol,^16,17^ hexanal,^18^ acetone,^19^ etc.^19^ Furthermore, previous studies by Yang et al.^20^ and Zaman et al.^12^ have demonstrated that CuO has a linear sub-Nernstian response to pH. The principle of pH sensitivity of metal oxides can be explained by binding theory: when metal oxide contacts with a solution, three types of surface charges are formed on the surface of metal oxide: negative (MO^–^), positive (MOH_2_^+^), and neutral (MOH).^21^ H^+^ and OH^–^ are attracted to negative and positive sites, respectively, leading to the formation of hydroxyl groups. For the RuO_2_, the reaction involving H^+^ ions is described in eq 1, and for CuO, the reaction can be written as eq 4(2)
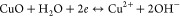
4In this paper, the properties and performance of binary pH-sensitive electrodes based on a mixture of ruthenium and copper oxides (RuO_2_–CuO electrodes) are presented. The electrodes described in this study were fabricated by the screen printing and investigated by means of potentiometry.

## Results and Discussion

### X-ray Diffraction Spectrum of the RuO_2_–CuO Electrodes

**Figure 2:**
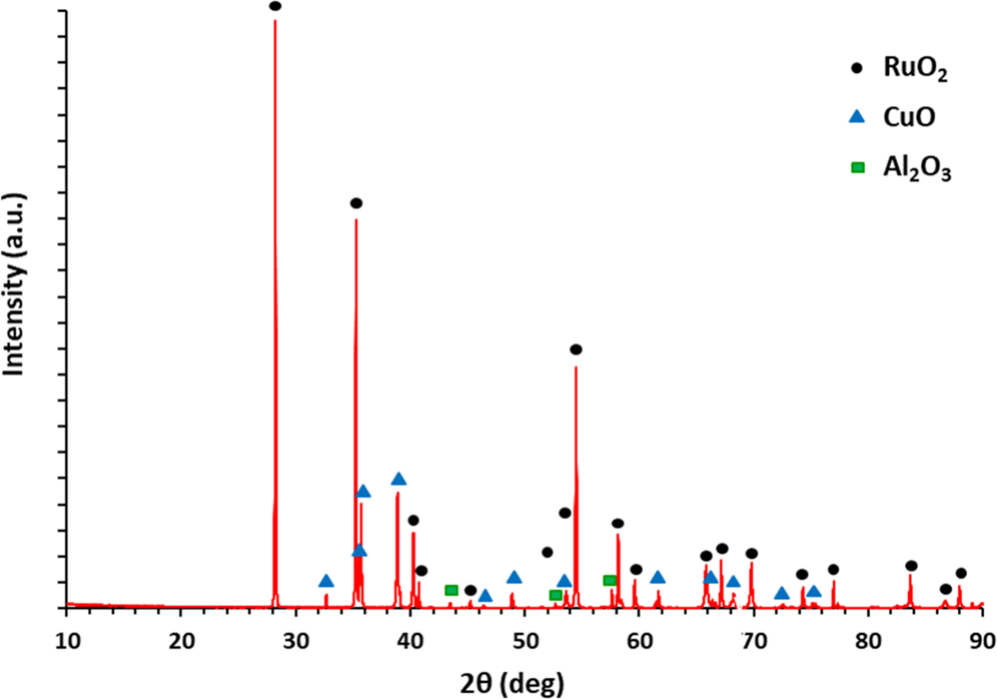
X-ray diffraction spectrum of the RuO_2_–CuO screen printed on the Al_2_O_3_ substrate and sintered at 900 °C. RuO_2_ and CuO were mixed at a 1:1 ratio.

In this study, the RuO_2_–CuO screen printing ink for the fabrication of the pH electrodes was made from commercially available RuO_2_ and CuO nanoparticle powders. The X-ray diffraction (XRD) spectrum of the of the screen-printed and sintered RuO_2_–CuO ink is presented in Figure 2. The results correlate with the rutile structure for the RuO_2_ (JCPDS 21-1172), the tenorite structure for CuO (JCPDS 98-009-2365), and the corundum structure for the Al_2_O_3_. Furthermore, evaluation of the peaks intensity revealed 46.8% of RuO_2_, 47.2% of CuO, and 6% Al_2_O_3_ in the sample and therefore verifying the 1:1 mixing ratio of the metal oxides. The particle size calculated from the Scherrer equation was equal to 107 ± 13 nm. This finding correlates well with those observed by Manjakkal et al. for screen-printing inks fabricated by mixing RuO_2_ with other oxides: Ta_2_O_5_, SnO_2_, and TiO_2_. For the RuO_2_–TiO_2_,^7^ RuO_2_–SnO_2_,^4^ and RuO_2_–Ta_2_O_5_,^8,22^ the average grain size confirmed by scanning electron microscopy (SEM) was 100–260, 90–210, and 180–520 nm, respectively.

### Sensitivity of the RuO_2_–CuO Electrodes

**Figure 3:**
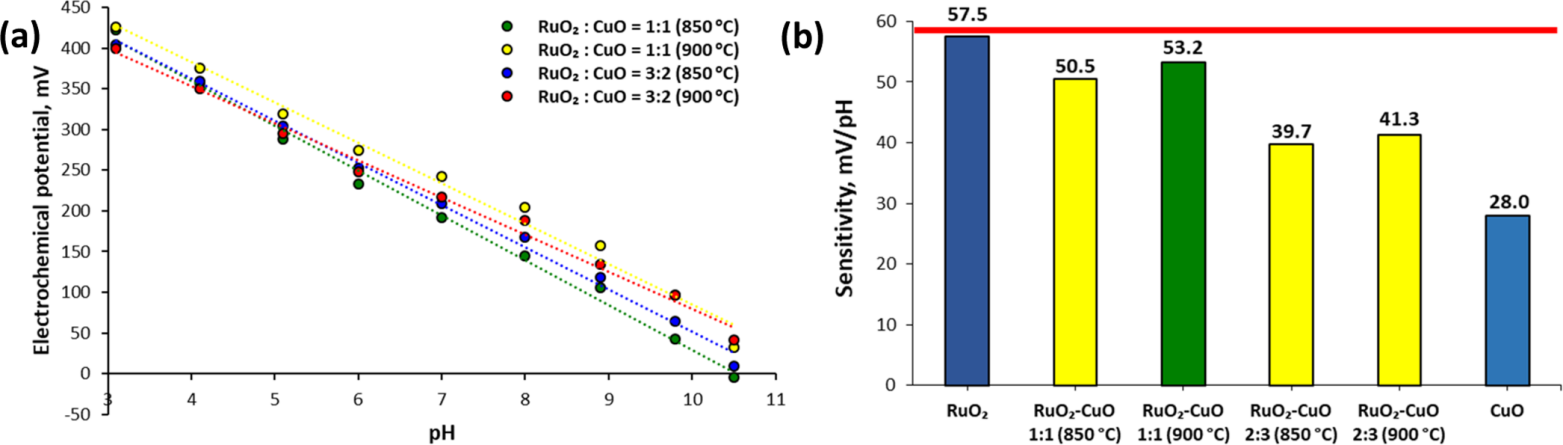
Among the fabricated electrodes, the RuO_2_–CuO electrodes with a RuO_2_:CuO ratio of 1:1 sintered at 900 °C (green) showed the best sensitivity. Graph (a) presents dependencies of the electrochemical potential of the fabricated RuO_2_–CuO electrodes (*Y*-axis) on pH (*X*-axis). Graph (b) presents the normalized sensitivity (*Y*-axis) of the fabricated RuO_2_–CuO electrodes compared to those of RuO_2_ (purple) and CuO (blue) electrodes and theoretical sensitivity (red line). Red horizontal lines indicate the deviation of 5 mV/pH from the theoretical Nernstian response. The RuO_2_–CuO electrodes with a RuO_2_:CuO ratio of 1:1 sintered at 900 °C (green) were selected for further investigation. The data on the CuO electrodes were taken from ref.^12^

Screen printing is a cost-effective technique that is suitable for the mass production of all-solid-state electrodes.^1,23^ Even though multiple parameters can be altered when depositing layers by the screen printing (e.g., changing the types of binders and solvents and introducing additives), in our study, the following was investigated: (i) ratio of RuO_2_ and CuO in the paste and (ii) sintering temperature. To determine the best RuO_2_-to-CuO ratio and sintering temperature, the fabricated electrodes were evaluated from the point of their sensitivity to pH changes. The results of the sensitivity measurement are presented in Figure 3 and Table S.1. The fabricated electrodes with a RuO_2_ to CuO ratio of 1:1 sintered at 900 °C showed pH sensitivity similar to the sensitivity observed for RuO_2_ electrodes and close to the theoretical sensitivity. Hence, these electrodes were selected for further study. For the RuO_2_–CuO electrodes with a greater amount of CuO, the sensitivity dropped by more than 10 mV/pH. This can be attributed to the lower sensitivity of CuO toward H^+^ ions: Zaman et al.^12^ previously reported sensor-based CuO nanoflowers that exhibited a near-Nernstian response of 28 mV/pH. In the study by Yang et al.,^20^ an extended gate field effect transistor-based pH sensor that incorporated CuO nanowires showed pH sensitivity of 18.4 mV/pH. Furthermore, lower pH sensitivity for a lower concentration of RuO_2_ correlates well with finding on electrodes fabricated from mixtures of RuO_2_ with Ta_2_O_5_, TiO_2_, and SnO_2_.^4,8,22^ Nevertheless, for RuO_2_–CuO electrodes with a RuO_2_ to CuO ratio of 1:1, the pH sensitivity was closer to that of RuO_2_ electrodes. This finding correlates well with the study by Manjakkal et al.^8^ The author reported a pH sensor fabricated from a mixture of RuO_2_ with tantalum(V) oxide (Ta_2_O_5_) mixed at RuO_2_ to Ta_2_O_5_ ratios of 7:3 and 3:7. The sensitivity of the RuO_2_–Ta_2_O_5_ electrodes mixed at 7:3 ratio was equal to 56.17 mV/pH, whereas the sensitivity of the RuO_2_–Ta_2_O_5_ electrodes mixed at 3:7 was equal to 35.3 mV/pH. Since the sensitivity of the RuO_2_–CuO electrodes with a RuO_2_ to CuO ratio of 1:1 sintered at 900 °C was close to the theoretical Nernstian sensitivity, other RuO_2_ to CuO ratios were not investigated.

### Comparison of the RuO_2_–CuO Electrodes to the RuO_2_ Electrodes

For the selected RuO_2_–CuO electrodes with a RuO_2_ to CuO ratio of 1:1 sintered at 900 °C, the remaining electrochemical characteristics were investigated and compared to those of a conventional glass electrode and RuO_2_ electrodes. The characteristics of the electrodes are presented in Table 1. The fabricated RuO_2_–CuO electrodes showed good linearity (*R*^2^ ∼ 0.990) and *E*^0^ values similar to those of RuO_2_ electrodes. For Nafion-covered RuO_2_ and RuO_2_–CuO electrodes, the electrochemical characteristics remained close to those of unmodified electrodes with slightly higher hysteresis and drift values since more time was required for ions to diffuse to the surface of the RuO_2_ layer through the Nafion membrane.^5,24^ Given that the Nafion membrane does not affect the performance of the RuO_2_–CuO electrodes, Nafion-covered electrodes were investigated for pH measurement in food samples.

**Table 1:** Electrochemical Characteristics of the Glass Electrode and RuO_2_-Based pH Electrodes

electrode type	sensitivity, mV/pH	*E*^0^, mV	*R*^2^	hysteresis A, mV	hysteresis B, mV	drift, mV/h
RuO_2_	57.5 ± 2.3	566.3 ± 55.3	0.979	3 ± 2	5 ± 2	0–5
RuO_2_–Nf	57.0 ± 0.7	684.1 ± 2.3	0.997	11 ± 1	17 ± 9	0–15
RuO_2_–CuO	54.3 ± 6.4	587.8 ± 15.6	0.989	5 ± 2	15 ± 2	0–5
RuO_2_–CuO–Nf	53.2 ± 1.6	575.3 ± 5.5	0.990	5 ± 2	20 ± 3	0–15
glass	58.8 ± 3.4	705.7 ± 36.8	0.997	10 ± 2	12 ± 3	0–5

### Cross-Sensitivity toward Interfering Ions

**Figure 4:**
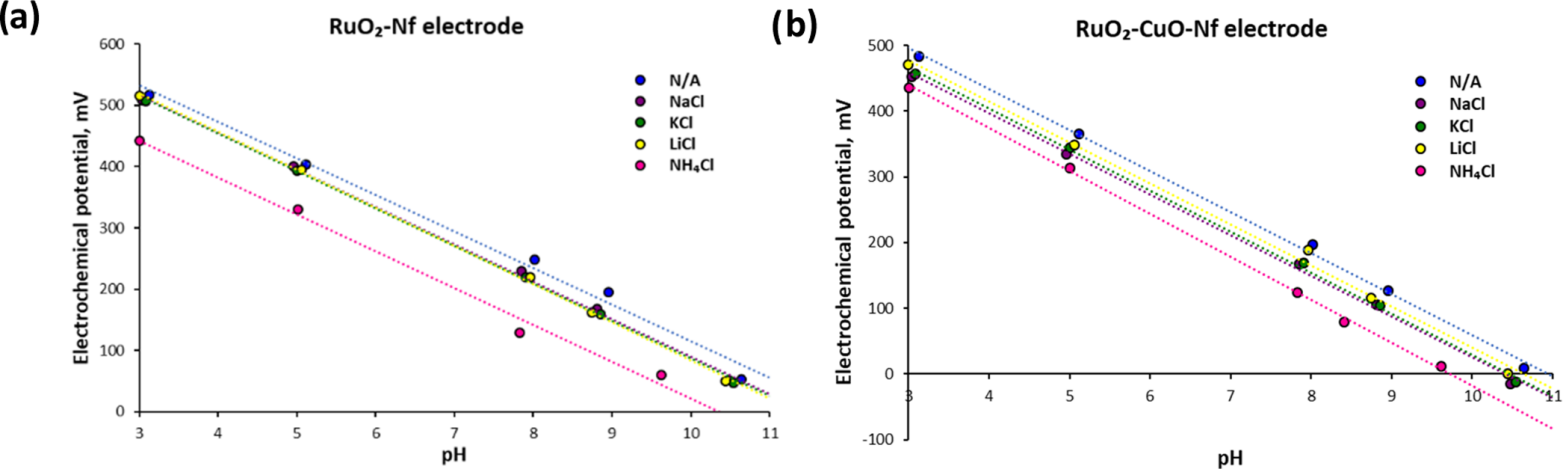
Electrochemical potential (*Y*-axis) of the fabricated RuO_2_–Nf (a) and RuO_2_–CuO–Nf (b) electrodes in the presence of Na^+^ (purple), K^+^ (green), Li^+^ (yellow), and NH_4_^+^ (pink). The ammonium ion influenced the *E*^0^ value the most.

Single-charged cations, such as Na^+^, K^+^, Li^+^, and NH_4_^+^, can interfere with precise pH measurement. To study the influence of these cations on the performance of the fabricated electrodes, the sensitivity of the fabricated Nafion-modified electrodes was determined in their presence. The results are presented in Figure 4 and Table 2. The pH sensitivity of the fabricated electrodes was not affected by the presence of the studied cations: the largest deviation was observed for the RuO_2_–CuO–Nf electrode in the presence of NH_4_^+^ ions and was equal to 2.9 mV/pH. Nevertheless, the RuO_2_–Nf and RuO_2_–CuO–Nf electrodes showed good linearity with *R*^2^ values above 0.991. The drop in *E*^0^ values observed for both RuO_2_–Nf and RuO_2_–CuO–Nf electrodes in the presence of NH_4_^+^ ions can be due to the decreased conductivity of Nafion membrane caused by the reaction between the NH_4_^+^ and SO_3_^–^ groups in Nafion backbone.^25^

**Table 2:** Characteristics of the RuO_2_–Nf and RuO_2_–CuO–Nf Electrodes in the Presence of the Interfering Ions

	RuO_2_–Nf			RuO_2_–CuO–Nf		
	sensitivity, mV/pH	*E*^0^, mV	*R*^2^	sensitivity, mV/pH	*E*^0^, mV	*R*^2^
no added salts	59.6	711.2	0.991	62.4	684.1	0.998
NaCl	60.9	699.9	0.998	61.9	644.2	0.998
KCl	61.2	698.9	0.999	62.4	654.4	0.999
LiCl	62.4	707.1	0.999	62.4	664.0	0.995
NH_4_Cl	60.1	622.9	0.991	65.3	634.8	0.999

### pH of Water Samples

The fabricated electrodes showed good performance in real-life water samples (Table 3). The average measurement accuracy was 0.23 and 0.05 pH units for the RuO_2_–Nf and RuO_2_–CuO–Nf electrodes, respectively. The fabricated electrodes exhibited a response similar to the conventional pH meter and a glass electrode with the maximum deviations of 0.36 and 0.12 pH units observed for RuO_2_–Nf and RuO_2_–CuO–Nf electrodes, respectively. Furthermore, all the fabricated electrodes showed good uniformity of the measured pH value (STD < 0.05 pH units).

**Table 3:** pH Values Measured with a Conventional Glass Electrode, RuO_2_–Nf and RuO_2_–CuO–Nf Electrodes in Different Water Samples

water sample	pH	glass electrode	RuO_2_–Nf	RuO_2_–CuO–Nf
tap water	7.7	7.69 ± 0.02	8.06 ± 0.01	7.68 ± 0.05
river water	7.34	7.35 ± 0.04	7.2 ± 0.05	7.35 ± 0.03
pond water	7.18	7.17 ± 0.02	6.99 ± 0.05	7.06 ± 0.04
seawater	7.54	7.55 ± 0.02	7.3 ± 0.01	7.5 ± 0.03

### pH of Food Samples

The performance of the solid-state pH electrodes in food samples can be different from their performance in diluted water samples or buffers due to a more complex composition or higher density. Even for measurement with a conventional glass electrode, adjustments should be made for proper pH measurement.^26^ The literature on the application of solid-state pH electrodes is scarce; to our knowledge, there are only a few articles. In 2008, Liao and Chou^27^ presented their working electrode, consisting of a RuO_2_ film sputtered on top of a silicon wafer. Their electrodes exhibited pH differences of 0.14 and 0.50 pH units for coke and milk, respectively. In 2015, Manjakkal et al.^4^ reported a screen-printed RuO_2_–SnO_2_ WE that was tested in lemon juice and showed a pH difference from a conventional glass electrode of 0.21 pH units. In 2018, Xu et al.^28^ reported their potentiometric system, consisting of a printed circuit board with two electrodes attached to it from the opposite sides: a sputtered antimony film on a copper substrate modified with a Nafion membrane as the WE and Ag|AgCl modified with a graphene-chitosan membrane as the RE. The reported electrodes showed pH differences of 0.19 and 0.11 pH units for coke and vinegar, respectively. Furthermore, Li et al.^29^ reported their potentiometric system utilizing poly(ethylene terephthalate)-covered indium tin oxide as the WE and Ti/Au/Ag/AgCl covered with a porous poly(vinyl butyral) membrane ion-selective field-effect transistor as the RE. For their electrodes, the pH difference was above 0.50 pH units in all the studied samples (coke, orange juice, beer, milk, etc.). The authors suggested that the pH difference from a conventional glass electrode can be due to the interference of proteins, organics, and additives in the beverages. Lonsdale et al.^30^ published their results on a WE that consisted of a RuO_2_ film sputter-deposited on an alumina substrate and modified with a sputtered Ta_2_O_5_ layer and drop-casted Nafion membrane. The electrodes showed excellent performance with pH differences not exceeding 0.08 pH units for the investigated samples, which included coke, beer, and milk. In 2020, Hu et al.^31^ reported a potentiometric pH sensor based on a graphite electrode modified with tryptophan residues. The sensor exhibited a sensitivity of 52 mV/pH and a deviation from the CGE of 0.15 pH units when used in milk and coke. Another article published in 2020 by Vivaldi et al.^32^ presented a screen-printed gold electrode modified with an indoaniline derivative as a pH-sensitive material. The sensor had a Nernstian response (56 mV/pH) and showed a deviation from the CGE of about 0.4 pH units when used in orange juice, milk, and tea.

**Figure 5:**
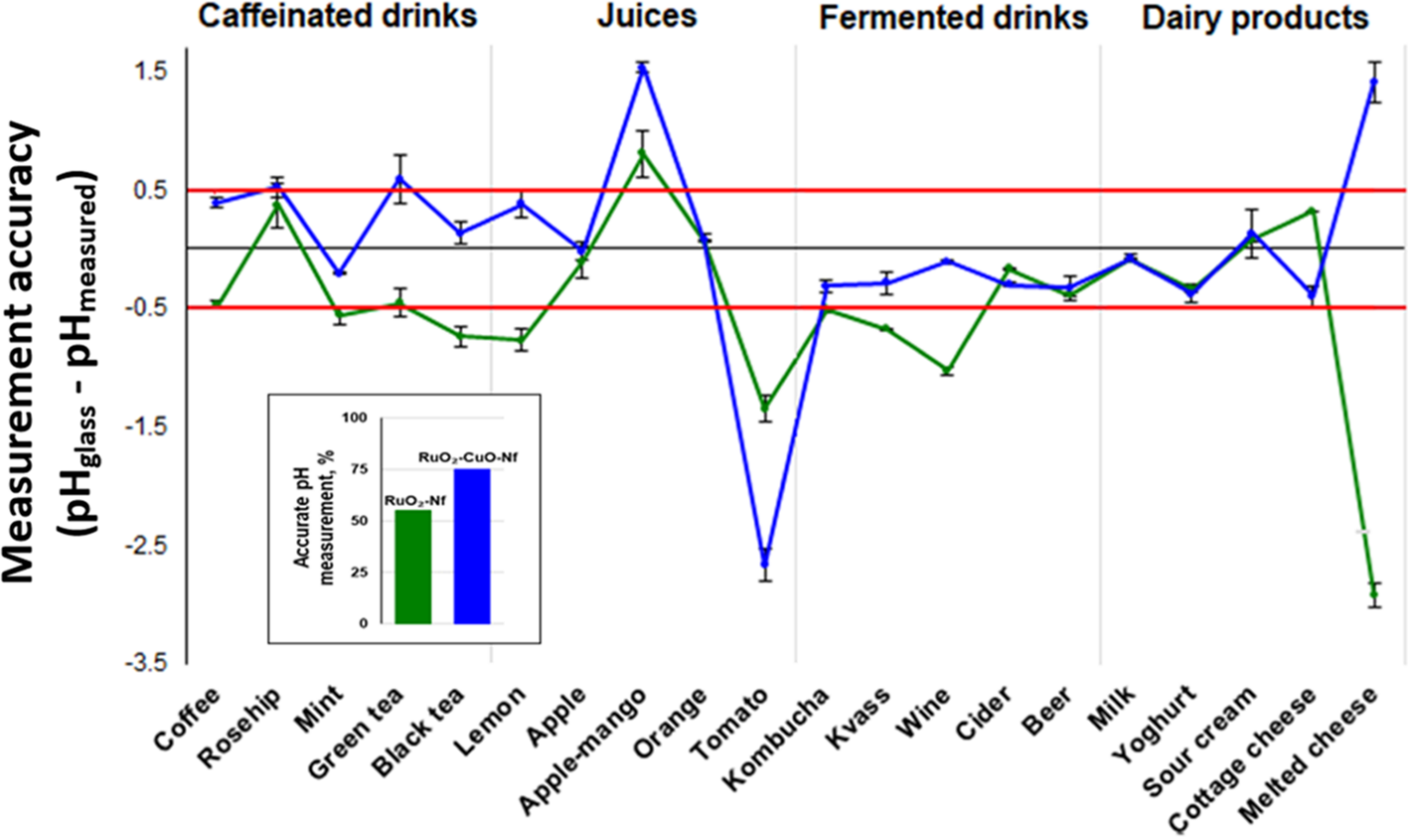
RuO_2_–CuO–Nf electrodes (blue) showed less scattered pH values measured for two electrodes in parallel compared to RuO_2_–Nf electrodes (green). Red horizontal lines (c) indicate the corridor or minimum (−0.5) and maximum (0.5) accepted errors. The accuracy of the electrodes was evaluated as a probability to measure the pH with the difference from the conventional glass electrode not exceeding 0.5 pH units. For the RuO_2_–Nf electrodes, the error exceeded 0.5 pH units in 45% of all measurements, and for RuO_2_–CuO–Nf electrodes, the error exceeded 0.5 pH units in only 25% of all measurements.

The results of the pH measurements with the fabricated RuO_2_–Nf and RuO_2_–CuO–Nf electrodes are presented in Figure 5 and Table S.2. The ±0.5 pH units were used as a reference margin.

In caffeinated drinks, the fabricated electrodes showed a significant difference from the pH values measured with a conventional glass electrode. For the RuO_2_–Nf electrode, the pH difference exceeded 0.5 pH units, making these electrodes unsuitable for pH measurement in tea or coffee samples. The RuO_2_–CuO–Nf electrodes showed better performance: the average pH difference was 0.36 pH units, with a pH difference exceeding 0.5 pH units only for the green tea sample (0.59 pH units).

The difference in the RuO_2_–Nf and RuO_2_–CuO–Nf electrode performance is more noticeable in the juice samples. Both electrode types showed errors exceeding 0.5 pH units in samples of higher density and thickness. Fruit juices contain ascorbic acid (reducing agent) that negatively affects the performance of metal oxide electrodes.^30^ Furthermore, the viscosity of the samples can negatively affect the potential of a metal oxide electrode.^33^ A more detailed study of this phenomenon is necessary and will be addressed in our future work.

The performance of the fabricated electrodes in fermented drinks was more accurate, with the RuO_2_–CuO–Nf electrodes showing an average pH difference of −0.27 pH units.

The investigation of the performance of the fabricated electrodes in dairy products revealed that both electrode types are suitable for pH measurement even in products with higher density. The fabricated electrodes only failed to measure the pH in melted cheese: the pH difference exceeded 1.4 pH units in both cases. For the melted cheese, the viscosity of the sample could have been the problem. In the dairy industry, measuring the pH of samples is a challenge. Usually, a homogenate is prepared by blending with water, and then, the pH of the homogenate is measured. In this study, we attempted to measure the pH of the product and not homogenate, thus, setting a challenging task. The challenges of measuring the pH in viscous samples correlate well with the findings of Chawang et al.,^33^ where authors have demonstrated that the viscosity of starch significantly influenced the measured potential of the iridium oxide electrode.

Furthermore, it is worth mentioning that the response time (time needed for an electrode to reach stable potential) in caffeinated drinks did not exceed 90 s for either electrode type. In the case of fermented drinks, apple and lemon juices, milk, and yoghurt, the time to reach stable potential did not exceed 5 min. For the samples of thicker texture, such as apple-mango, orange, and tomato juices, sour cream, cottage, and melted cheese, the measurement was conducted for almost 10 min.

Overall, the RuO_2_–Nf electrodes failed to accurately measure pH in 9 out of 20 investigated samples, while RuO_2_–CuO–Nf electrodes failed only in 5 out of 20 samples; thus, an almost twofold improvement in the performance of the pH electrodes was observed.

## Conclusions

In conclusion, electrodes based on binary oxide RuO_2_ and CuO fabricated by a screen-printing technique were tested for pH measurement for the first time. The application of the electrodes in real-life food samples was possible due to the coverage of the electrodes with a Nafion protective membrane. The RuO_2_–CuO–Nf electrodes surpass RuO_2_–Nf electrodes in potentiometric pH measurement not only from the point of cost-effectiveness but also the overall performance in real-life samples. The proposed electrodes aim to replace fragile glass electrodes in the pH measurement of food samples. Since the reported electrodes are physically durable, they can be of interest to food researchers not only in research but also in industrial pH measurement. Binary electrodes are equal to both conventional glass electrodes and previously reported RuO_2_ electrodes from the point of view of the electrochemical characteristics. Furthermore, RuO_2_–CuO pH electrodes covered with a protective Nafion membrane are compatible with pH measurements of common beverages and dairy products. Significant error, exceeding 0.5 pH units, was observed only when measuring specific juices and cheese. Apparently, the texture of the sample, as well as its composition, can affect the performance of the screen-printed RuO_2_-based electrode. In our future work, we plan to further investigate the influence of the abovementioned factors.

## Materials and Methods

### Fabrication of RuO_2_ Electrodes

**Figure 6:**
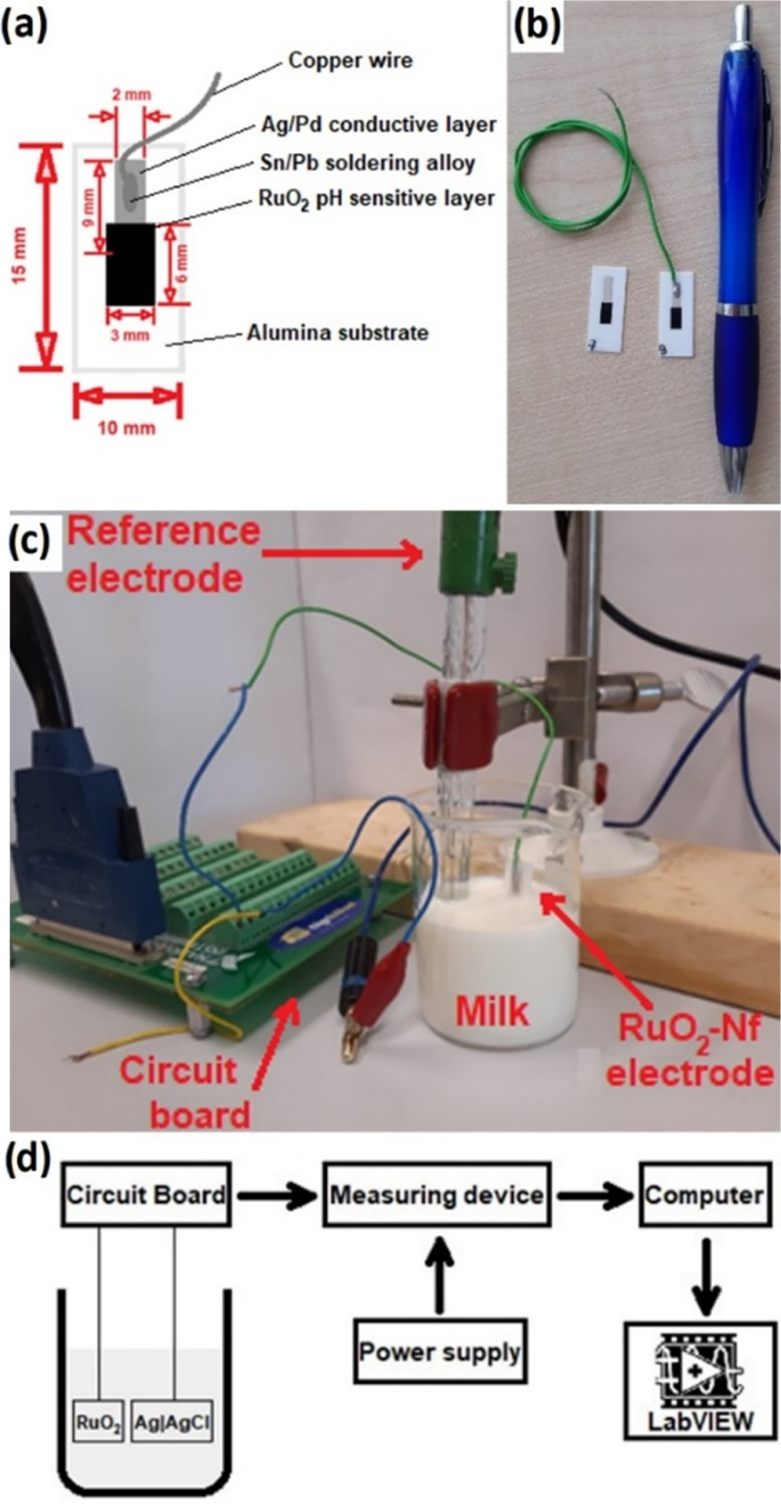
The RuO_2_ electrodes were fabricated by screen printing a conductive Ag/Pd layer and pH-sensitive RuO_2_ layer on an alumina substrate (a). A copper wire was connected to the conductive layer by soldering with a Pb/Sn alloy. In Figure (b), a pen is placed next to the fabricated sensors for comparison. For the electrochemical measurement, a fabricated electrode and a standard glass reference electrode were placed into a sample solution (c). In Figure (d), the scheme of the measuring setup is presented: the RuO_2_ pH-sensitive electrode and Ag|AgCl reference electrode were connected to the measuring device via the circuit board. The measuring supply was powered with an input voltage of 12 V. The data were registered and monitored using the LabView program.

The electrodes were fabricated similarly to previously described methods.^34^ Briefly, two layers were deposited on an alumina (Al_2_O_3_) substrate by the screen printing: a conductive layer and a pH-sensitive layer. A conductive layer of Ag/Pd thick film paste (9695, Electro-Science Laboratories, King of Prussia, Pennsylvania) was printed first, and a pH-sensitive layer of commercially available RuO_2_ paste (10 kΩ/sq, 3914, Electro-Science Laboratories, King of Prussia, Pennsylvania) was printed second. Furthermore, the RuO_2_ layer was printed in such a way that it would partly overlap the conductive Ag/Pd layer. The substrates were dried at 120 °C for 15 min and consequently sintered at 850 °C for 1 h after the first printing step and at 900 °C for 1 h after the second printing step. After cooling the substrate, a copper wire was connected to the Ag/Pd layer by soldering with a Sn/Pb alloy. Finally, a protective layer of silicone rubber (DOWSIL 3140 RTV Coating, Dow Chemical Company, Midland, Michigan) was used to cover the conductive layer and the electric contact. The dimensions of the fabricated RuO_2_ electrodes are presented in Figure 6a,b. The screen-printed RuO_2_ electrodes were previously characterized by Manjakkal et al.^35−37^

### Fabrication of RuO_2_–CuO Electrodes

The RuO_2_–CuO electrodes were fabricated similarly to the RuO_2_-based electrodes previously described by Manjakkal and co-workers.^4,7,8^ The only difference was in the paste used for the deposition of the pH-sensitive layer. For the fabrication of the RuO_2_–CuO electrodes, the RuO_2_–CuO paste was prepared before screen printing. Anhydrous RuO_2_ (99.9% pure, Sigma-Aldrich, USA) and CuO (average particle size 40–80 nm, 99.9% pure, Chempur, Germany) were mixed in an agate mortar. Ethylcellulose (analytical grade purity) and terpineol (anhydrous, Fluka Analytical) were added to the mortar as binders for the paste. The oxides were mixed for 20 min to achieve optimal consistency of the paste.

Two RuO_2_/CuO ratios were investigated to determine what part of RuO_2_ can be successfully substituted with CuO without compromising the electrode performance: 1:1 and 2:3. All the other parameters of the fabrication remained the same.

Two different temperatures were used to investigate the influence of the sintering temperature on the properties of the RuO_2_–CuO electrodes: 850 and 900 °C. The sintering temperature was previously demonstrated to not affect RuO_2_ electrodes.^3^ All the other parameters of the fabrication remained the same.

The crystalline structure of the RuO_2_–CuO screen printing ink was conducted by the X-ray diffraction (XRD) analysis using a Empyrean diffractometer (Malvern Pananalytical, U.K.). The copper target (1.54 Å) was used to record the intensity of the diffraction in the range of 5···90° 2θ. Phase identification was performed according to the International Center for Diffraction Database (ICDD). Crystallite size was calculated from the Scherrer equation.^38^ Scanning electron microscopy (SEM) and transmission electron microscopy (TEM) were not performed in this study.

### Deposition of the Nafion Membrane

To make the fabricated electrodes suitable for measurement in dairy products, the fabricated electrodes were covered with a Nafion protective membrane by the drop-casting technique. The methodology for the Nafion membrane deposition was previously reported elsewhere.^34^ Briefly, 10 μL of 5% solution of Nafion in a mixture of lower aliphatic alcohols and water (Nafion 117, Sigma-Aldrich, USA) was applied to cover the pH-sensitive area of the fabricated electrodes. Next, the electrodes were dried in a laboratory incubator (BD 53, Binder, Germany) at 80 °C for 2 h. The Nafion solution was pipetted on the electrodes and dried in the laboratory incubator two more times, thus creating three layers of the Nafion membrane. After the last layer was dried in the laboratory incubator, the electrodes were left to air-dry at room temperature overnight. The RuO_2_ and RuO_2_–CuO electrodes modified with Nafion were named RuO_2_–Nf and RuO_2_–CuO–Nf, respectively.

### Setup for Potentiometric Measurement

All the measurements were performed in an electrochemical cell, as presented in Figure 6c,d. One of the fabricated electrodes (RuO_2_, RuO_2_–CuO, RuO_2_–Nf, or RuO_2_–CuO–Nf) and a standard glass ion-selective Ag|AgCl (RL-100, HYDROMET, Poland) reference electrode were connected to the measuring device (Data Acquisition (DAQ) device, USB-6259, National Instruments, USA) through a circuit board via galvanic connections. The measuring device was powered by a high-performance digital power supply (E3631A, Agilent, USA) with an input voltage of 12 V. The potential difference between a fabricated and the reference electrode was monitored and registered with the use of the LabVIEW program (National Instruments, USA).

### Determination of Electrochemical Characteristics

The following characteristics were determined to evaluate the electrode performance: sensitivity and linearity, hysteresis and drift effects, and cross-sensitivity to interfering ions. The abovementioned characteristics were measured for a conventional glass electrode (HI1053P, Hanna Instruments, USA) as well. All the measurements were conducted in triplicate for 2 electrodes of the same kind if not specified otherwise.

All the electrochemical characteristics of the fabricated electrodes were evaluated after one month of conditioning in water. This preliminary condition is necessary for an electrode to reach stable working conditions.^39^

*The sensitivity* of the fabricated electrodes were determined by calibrating them against buffer solutions. Buffer solutions in the pH range of 3.0–11.0 were used. Buffers were freshly prepared from anhydrous salts (Sigma-Aldrich, Massachusetts) according to the procedure described by Dawson et al.^40^ The pH of the buffers was determined with a conventional pH meter (Seven2Go Advanced Single-Channel Portable pH Meter, Mettler Toledo, Switzerland). To calibrate an electrode, the potential of the electrode was determined in several buffer solutions 90 s after immersing the electrode into a buffer solution. The values of the electrode potential (*Y*-axis) were plotted against the pH of the buffers (*X*-axis), and the sensitivity was determined as a slope of the function *E* = *f*(pH) by the method of least squares. *E*^0^ was determined by extrapolating the function to the intersection with the *Y*-axis.

The sensitivity was determined for all the fabricated electrode types (RuO_2_, RuO_2_–CuO, RuO_2_–Nf, and RuO_2_–CuO–Nf) to evaluate whether the Nafion membrane is suitable for RuO_2_–CuO electrodes. Since the sensitivity of the RuO_2_–CuO electrodes with and without Nafion was similar (as for RuO_2_ electrodes) and the electrodes without a Nafion protective layer ceased working in food samples, all of the following characteristics were evaluated for the RuO_2_–Nf and RuO_2_–CuO–Nf electrodes only.

The hysteresis (mV) is a characteristic of an electrode that is observed when the electrode has different potential values in the same media due to the previous electrode’s exposure to a solution of different pH values. Hysteresis is associated with changes in the composition of the double layer on the surface of the electrode. Hysteresis of the fabricated electrodes was determined by exposing the electrodes to the buffer solutions in a loop manner. Two loops were investigated separately: an acidic loop (pH values of 3-5-7-5-3) and a basic loop (pH values of 11-9-7-9-11). A fabricated electrode and the reference electrode were placed into a buffer solution, and the potential was recorded 5 min after the submersion of the electrodes into the buffer. Then, the electrodes were rinsed with distilled water, gently tapped with a paper towel, and placed into the next buffer solution. Hysteresis was determined as the difference in the potential values of the electrode at pH 3.

The drift of the potential of an electrode (mV/h) is defined as a slow nonrandom change in the reading of an electrode with time. The drift of the electrode potential is associated with the diffusion of H^+^ ions.^41^ The drift rate of the fabricated electrodes was determined by recording the potential of an electrode for 2 h and calculating the average difference (per hour) in the potential values of an electrode at the beginning of the measurement and after 2 h of continuous potential measurement.

The presence of some of the compounds in the sample can affect the performance of a solid-state electrode.^3,4,8^ The interference of ions with the performance of the fabricated electrodes was evaluated by determining the sensitivity of the electrodes in the presence of specific anions and cations. Buffer solutions additionally containing the chlorides of Li^+^, Na^+^, K^+^, and NH_4_^+^ at a concentration of 0.1 M were prepared (other cations were not investigated since the Nafion membrane allows only small ions to pass through^25^). The potential of the electrode was determined in the buffer solutions 90 s after immersing the electrode into each buffer.

The Electrical Impedance Spectroscopy (EIS) was not performed in this study; however, the capacitive characteristics of the RuO_2_–CuO electrodes are expected to be similar to those of RuO_2_–SnO_2_ previously described by Manjakkal et al.:^4^ RuO_2_–CuO electrodes are expected to have more capacitive nature than RuO_2_ electrodes. The Nyquist plot of RuO_2_ consists of a bigger semi-circular arc in low frequency range that is due to adsorption of ions on the surface of the electrode.^37^ For the CuO, the semi-circle which is observed in the higher frequency range and is due to the charge-transfer process of H^+^/OH^–^ ions at the CuO/solution interface.^14^

The stability and the reusability of the RuO_2_–Nf electrodes was reported in our previous works.^5,42^ Briefly, the stability of the RuO_2_ electrodes was evaluated by monitoring the sensitivity over the course of 7 weeks. The sensitivity was changing during first 3 weeks and then remained at the same value.^5^ Furthermore, the RuO_2_–Nf electrodes were tested for 1 h-long measurement in milk and exhibited performance similar to the conventional glass electrode.^5^ The RuO_2_–Nf electrodes were shown to be reusable by renewing the Nafion membrane.^42^ The stability of the RuO_2_–CuO–Nf electrodes is similar to the RuO_2_–Nf electrodes.

### Measurement in Real-Life Samples

The pH values of the samples were determined by two-point calibration, which is widely used in laboratory practice.^26,43,44^ For this purpose, commercially available certified buffers (Certipur, Merk, New Jersey) with pH values of 4 and 7 were used. The RuO_2_–Nf or RuO_2_–CuO–Nf electrode was placed into a buffer solution of pH 7, and readings of the voltmeter were recorded for 5 min. Then, the electrode was rinsed with Milli-Q water and placed into a buffer solution of pH 4, and the potential was again measured for 5 min. The electrode was rinsed with Milli-Q water again and placed into a sample. The potential of the electrode was registered 5 min after placing the electrode into the sample. All the measurements were made in triplicate for two identical electrodes.

**Figure 7:**
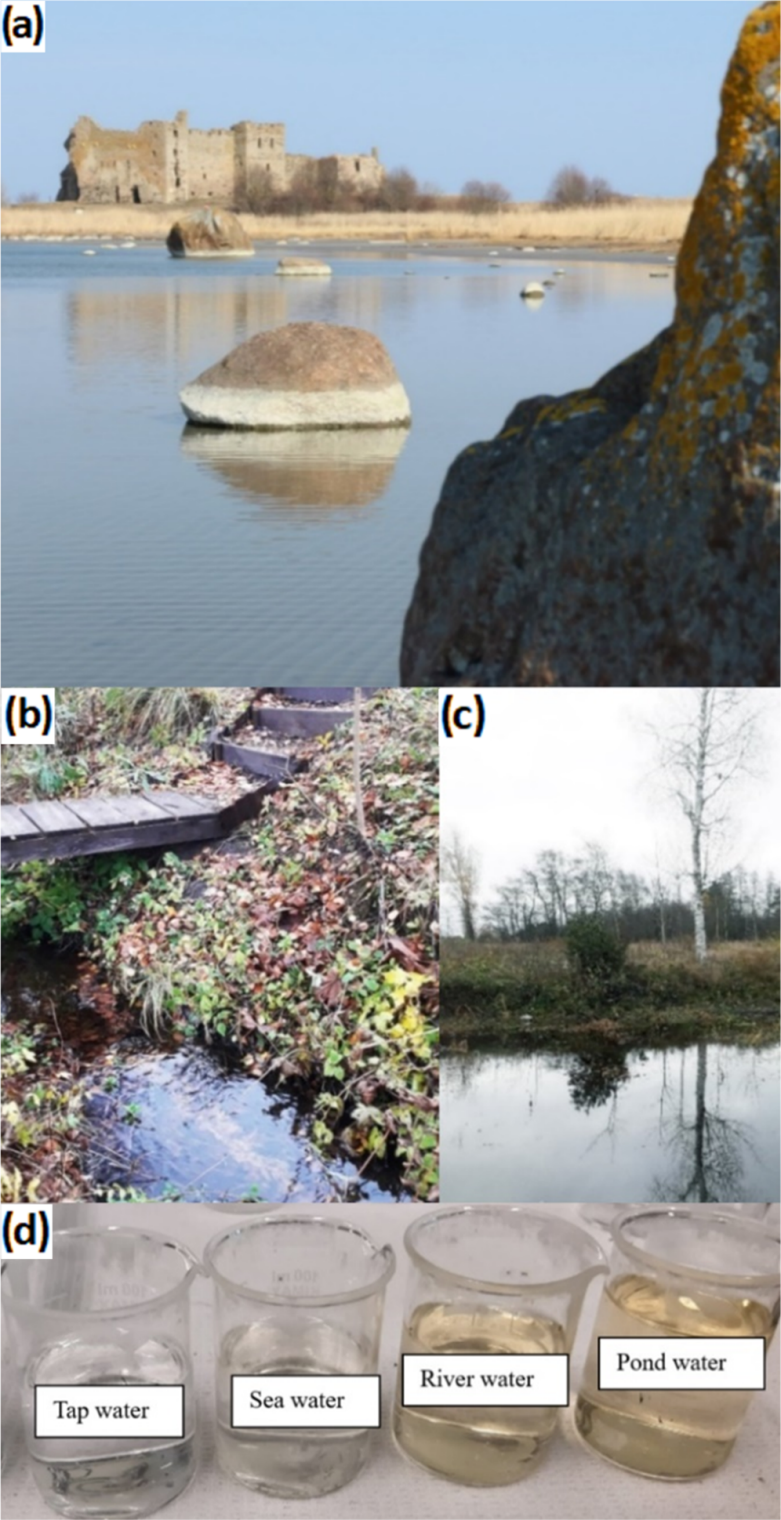
Photos of the places where water samples were collected: (a) Kunda bay of Baltic sea, (b) pond, and (c) river in Toolse village, (d) side-by-side comparison of collected water samples.

The performance of the fabricated electrodes was evaluated as the measurement accuracy determined as the difference in pH readings between a fabricated electrode (pH_measured_) and the pH meter (pH_glass_) on the basis of the following formula
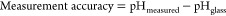
5Water samples from different sources (Table 4 and Figure 7) were collected to evaluate the performance of the fabricated electrodes. The seawater was collected at the Kunda bay of the Baltic sea 3 meters from the shore. The pond water was collected from the surface of a small pond near Toolse village, Haljala vald, Estonia. All of the samples were stored at 4 °C and allowed to warm up to room temperature prior to any measurement. The pH value of the collected samples was measured with a conventional pH meter.

**Table 4:** Water Samples Used for the Investigation of the Performance of the Fabricated Electrodes

sample	pH	collection place
tap water	7.70	lab sink
river water	7.34	river near Toolse village, Estonia
pond water	7.18	pond near Toolse village
seawater	7.54	Kunda bay near Toolse village

All the food samples (Table 5) were purchased from a local grocery store. The samples were brought to room temperature (22 °C) prior to any measurements. A conventional pH meter was used to determine the pH values of the food samples.

**Table 5:** Food Samples Used for the Investigation of the Performance of the Fabricated Electrodes

sample	pH	manufacturer	sample	pH	manufacturer
*caffeinated drinks*			*fermented drinks*		
Rosehip tea	3.21	Herba, Germany	Kombucha	3.67	GUTsy, Portugal
coffee	5.08	Jacobs, Germany	Kvass	3.61	A. Le Coq, Estonia
mint tea	6.83	Herba, Germany	wine	3.42	Mirabeau, France
green tea	6.97	Tetley, U.K.	cider	3.22	A. Le Coq, Estonia
black tea	7.31	Tetley, U.K.	beer	4.38	Heineken, Netherlands
*juices*			*dairy products*		
lemon juice	2.55	ICA, Italy	milk	6.68	Valio, Estonia
apple juice	3.06	A. Le Coq, Estonia	yogurt	4.36	Valio, Estonia
apple-mango juice	3.80	A. Le Coq, Estonia	sour cream	4.35	Valio, Estonia
orange juice	3.93	A. Le Coq, Estonia	cottage cheese	4.68	Valio, Estonia
tomato juice	4.31	A. Le Coq, Estonia	melted cheese	5.96	Valio, Estonia

## Supporting Information Available

Sensitivity values for the RuO_2_–CuO electrodes at different mixing ratios and sintering temperatures; pH values measured with a conventional glass electrode, RuO_2_–Nf and RuO_2_–CuO–Nf electrodes in different food samples (PDF)
