# Supplementary material for: Binary RuO2–CuO Electrodes Outperform RuO2 Electrodes in Measuring the pH in Food Samples
Source: ACS Omega. 2023 Mar 30;8(14):13275–84. doi: 10.1021/acsomega.3c00538 (PMC10099411; doi:10.1021/acsomega.3c00538)
Supplement: Supplementary file 1 — ao3c00538_si_001.pdf [file ao3c00538_si_001.pdf]

SUPPORTING INFORMATION

Binary RuO_2-CuO electrodes outperform RuO_2 electrodes in measuring the pH in food samples

Maryna Lazouskaya [a,b,*], Iuliia Vetik [a], Martti Tamm [b], Kiranmai Uppuluri [c], Ott Scheler [a]

[a] *School of Science, Department of Chemistry and Biotechnology, Tallinn University of Technology, Ehitajate tee 5, 19086 Tallinn, Estonia*
[b] *Center of Food and Fermentation Technologies (CFFT), Akadeemia tee 15a, 12618 Tallinn, Estonia*
[c] *Łukasiewicz Research Network–Institute of Microelectronics and Photonics, Krakow, Poland*

[*] *Corresponding author; Email: maryna.lazouskaya@taltech.ee*

Table S.1. Sensitivity of the RuO_2-CuO-Nf electrodes after one-month conditioning in water.

RuO_2:CuO ratio	Sintering temperature, °C	Sensitivity, mV/pH	E^0, mV	R^2
1:1	850	50.5 ± 4.5	554 ± 25.5	0.987
1:1	900	54.3 ± 6.4	587.8 ± 15.6	0.989
3:2	850	39.7 ± 12.0	420.4 ± 148.7	0.959
3:2	900	41.3 ± 4.3	470.0 ± 65.5	0.947

Table S.2. pH values measured with a conventional glass electrode, RuO_2-Nf and RuO_2-CuO-Nf electrodes in different food samples.

Sample	pH	Glass electrode	RuO_2-Nf	RuO_2-CuO-Nf
**Caffeinated drinks**				
Coffee	5.08	5.13 ± 0.01	5.60 ± 0.03	4.74 ± 0.05
Rosehip	3.21	3.18 ± 0.01	2.81 ± 0.19	2.66 ± 0.09
Mint	6.83	6.84 ± 0.02	7.40 ± 0.08	7.05 ± 0.01
Green tea	6.97	7.10 ± 0.10	7.56 ± 0.12	6.51 ± 0.20
Black tea	7.31	7.21 ± 0.04	7.95 ± 0.09	7.08 ± 0.09
**Juices**				
Lemon	2.55	2.44 ± 0.01	3.21 ± 0.09	2.06 ± 0.11
Apple	3.06	2.88 ± 0.01	2.99 ± 0.13	2.90 ± 0.08
Apple-mango	3.80	3.87 ± 0.01	3.07 ± 0.19	2.34 ± 0.04
Orange	3.93	4.04 ± 0.01	3.98 ± 0.06	3.97 ± 0.01
Tomato	4.31	4.33 ± 0.01	5.68 ± 0.11	7.00 ± 0.14
**Fermented drinks**				
Kombucha	3.67	3.90 ± 0.04	4.41 ± 0.02	4.21 ± 0.05
Kvass	3.61	3.87 ± 0.01	4.55 ± 0.01	4.16 ± 0.10
Wine	3.42	3.32 ± 0.01	4.35 ± 0.04	3.43 ± 0.02
Cider	3.22	3.46 ± 0.01	3.63 ± 0.01	3.76 ± 0.01
Beer	4.38	4.59 ± 0.02	4.98 ± 0.02	4.92 ± 0.10
**Dairy products**				
Milk	6.68	6.68 ± 0.01	6.77 ± 0.01	6.76 ± 0.03
Yoghurt	4.36	4.35 ± 0.00	4.69 ± 0.03	4.73 ± 0.08
Sour cream	4.35	4.31 ± 0.00	4.23 ± 0.02	4.18 ± 0.20
Cottage cheese	4.68	4.78 ± 0.01	4.46 ± 0.00	5.18 ± 0.09
Melted cheese	5.96	5.99 ± 0.01	8.91 ± 0.10	4.58 ± 0.17
